# Supplementary material for: Evidence Accumulation Rate Moderates the Relationship between Enriched Environment Exposure and Age-Related Response Speed Declines
Source: J Neurosci. 2023 Sep 13;43(37):6401–14. doi: 10.1523/JNEUROSCI.2260-21.2023 (PMC10500991; doi:10.1523/JNEUROSCI.2260-21.2023)
Supplement: Figure 2-1 — Bayesian linear regression model; the relationship between RT and cognitive reserve. Download Figure 2-1, DOCX file. [file ns-JN-RM-2260-21-s01.docx]

**Extended Data Figure 2-1:** Bayesian Linear Regression Model the relationship between RT and Cognitive Reserve.

| **Model Comparison** | | | | | | | | | | |
| --- | --- | --- | --- | --- | --- | --- | --- | --- | --- | --- |
| **Models** | | **P(M)** | | **P(M\|data)** | | **BF _M_** | | **BF _10_** | | **R²** |
| Null model |  | 0.250 |  | 0.077 |  | 0.251 |  | 1.000 |  | 0.000 |
| CRI Leisure + CRI Work + CRI Education |  | 0.250 |  | 0.380 |  | 1.837 |  | 4.917 |  | 0.266 |
| CRI Leisure + CRI Work |  | 0.083 |  | 0.317 |  | 5.104 |  | 12.311 |  | 0.263 |
| CRI Leisure |  | 0.083 |  | 0.110 |  | 1.358 |  | 4.269 |  | 0.154 |
| CRI Leisure + CRI Education |  | 0.083 |  | 0.074 |  | 0.874 |  | 2.860 |  | 0.190 |
| CRI Work |  | 0.083 |  | 0.022 |  | 0.253 |  | 0.873 |  | 0.065 |
| CRI Education |  | 0.083 |  | 0.011 |  | 0.121 |  | 0.421 |  | 0.020 |
| CRI Work + CRI Education |  | 0.083 |  | 0.009 |  | 0.102 |  | 0.358 |  | 0.067 |
