# Supplementary material for: Evidence Accumulation Rate Moderates the Relationship between Enriched Environment Exposure and Age-Related Response Speed Declines
Source: J Neurosci. 2023 Sep 13;43(37):6401–14. doi: 10.1523/JNEUROSCI.2260-21.2023 (PMC10500991; doi:10.1523/JNEUROSCI.2260-21.2023)
Supplement: Figure 2-2 — Frequency and duration of leisure activity engagement for those with high and low CRI Leisure (devised by median split). Download Figure 2-2, DOCX file. [file ns-JN-RM-2260-21-s02.docx]

**Extended Data Figure 2-2** Frequency and duration of leisure activity engagement for those with high and low CRI Leisure (devised by median split).

|  | Overall (n = 41) | | High CR-Leisure (n = 20) | | Low CR-Leisure (n = 21) | | Significant difference for proportion of time engaged |
| --- | --- | --- | --- | --- | --- | --- | --- |
|  | Percentage of People Engaged | Proportion of Life Engaged | Percentage of People Engaged | Proportion of Life Engaged | Percentage of People Engaged | Proportion of Life Engaged |  |
| **Activities with weekly frequency** |  |  |  |  |  |  |  |
| Reading newspapers and magazines | 90.2% (*n*=37) | 82.89% (32.25) | 100.0% (*n*=20) | 89.28% (26.81) | 81.0% (*n*=17) | 75.38% (37.08) | ns. |
| Housework (cooking, ironing, washing, etc) | 97.6% (*n*=40) | 85.61% (32.85) | 100.0% (*n*=20) | 89.71% (33.06) | 95.2% (*n*=20) | 81.50% (32.97) | ns. |
| Driving (not biking) | 95.1% (*n*=39) | 97.34% (14.39) | 95.0% (*n*=19) | 102.02% (5.84) | 95.2% (*n*=20) | 92.89% (18.41) | * |
| Leisure activities (sports, hunting, dancing, cards, bowling, etc) | 95.1% (*n*=39) | 53.58% (31.65) | 95.0% (*n*=19) | 64.97% (19.84) | 95.2% (*n*=20) | 42.76% (30.11) | * |
| **Using new technologies (digital camera, computer, internet, etc)** | **100.0% (*n*=41)** | **72.46% (31.12)** | **100.0% (*n*=20)** | **90.53% (24.51)** | **100.0% (*n*=21)** | **55.26% (27.00)** | ******* |
| **Activities with monthly frequency** |  |  |  |  |  |  |  |
| **Social activities (parties/going out with friends, local community events, etc)** | **87.8% (*n*=36)** | **51.90% (38.67)** | **90.0% (*n*=18)** | **75.17% (38.25)** | **85.7% (*n*=18)** | **28.64% (21.65)** | ******* |
| Cinema or theatre | 41.5% (*n*=17) | 48.25% (34.20) | 45.0% (*n*=9) | 66.79% (36.17) | 38.1% (*n*=8) | 27.38% (15.45) | ** |
| Gardening, handcraft, knitting, etc | 85.4% (*n*=35) | 79.81% (31.07) | 90.0% (*n*=18) | 78.58% (34.77) | 81.0% (*n*=17) | 81.11% (27.64) | ns. |
| Taking care of children or elderly | 63.4% (*n*=26) | 24.39% (13.43) | 60.0% (*n*=12) | 24.87% (13.84) | 66.7% (*n*=14) | 23.99% (13.57) | ns. |
| Volunteering | 73.2% (*n*=30) | 25.39% (18.38) | 80.0% (*n*=16) | 26.11% (23.82) | 66.7% (*n*=14) | 24.57%  (9.88) | ns. |
| Artistic activities (playing an instrument, painting, writing, etc) | 53.7% (*n*=22) | 52.26% (43.53) | 60.0% (*n*=12) | 66.41% (41.23) | 47.6% (*n*=10) | 39.68% (43.71) | ns. |
| **Activities with annual frequency** |  |  |  |  |  |  |  |
| **Exhibitions, concerts, conferences** | **82.9% (*n*=34)** | **59.35% (35.84)** | **100.0% (*n*=20)** | **76.36% (30.60)** | **66.7% (*n*=14)** | **35.06% (28.51)** | ******* |
| Holidays | 73.2% (*n*=30) | 49.82% (30.42) | 90.0% (*n*=18) | 60.91% (33.45) | 57.1% (*n*=12) | 33.20% (14.43) | ** |
| Reading books | 100.0% (*n*=41) | 87.69% (34.83) | 100.0% (*n*=20) | 99.13% (19.26) | 100.0% (*n*=21) | 76.81% (42.65) | * |
| **Activities with fixed frequency** |  |  |  |  |  |  |  |
| Pet care | 73.2% (*n*=30) | 53.14% (36.48) | 85.0% (*n*=17) | 58.91% (38.43) | 61.9% (*n*=13) | 45.60% (33.75) | ns. |
| Managing one’s bank account(s) | 100.0% (*n*=41) | 94.20% (21.20) | 100.0% (*n*=20) | 99.65% (13.98) | 100.0% (*n*=21) | 89.00% (25.59) | ns. |
| Having children | 82.9% (*n*=34) | 2.82  (.94) | 70.0% (*n*=14) | 2.64  (.93) | 95.2% (*n*=20) | 2.95  (.94) | ns. |

ns. *p*>.05; * *p*≤.05; ** *p*≤.01; *** *p*≤.001, only these values pass the Bonferroni threshold for significance.

*Note.* All values represent *M*(SD). Values reported for having children are the percentage of people who reported having children, and the mean number of children, respectively.
