# Supplementary material for: Evidence Accumulation Rate Moderates the Relationship between Enriched Environment Exposure and Age-Related Response Speed Declines
Source: J Neurosci. 2023 Sep 13;43(37):6401–14. doi: 10.1523/JNEUROSCI.2260-21.2023 (PMC10500991; doi:10.1523/JNEUROSCI.2260-21.2023)
Supplement: Figure 2-3 — The effects of CRIq Leisure on RT are statistically independent to the association between RT and CRI Education and CRI Occupation. To explore the association between the CRIq Leisure subscale with Education and Occupation, we ran correlational analyses between the four CRI subscales. First, the composite score was strongly associated with leisure, occupation, and education engagements (all r values > 0.65), p < 0.001). A robust association was also observed between the CRI subscales of Education and Occupation (r > 0.4, table below). Critically, however, we did not find evidence to suggest an association existed between the CRI Leisure subscale with CRI Education (r = 0.11, p = 0.48) or CRI Occupation (r = 0.18, p = 0.27). In fact, follow-up Bayesian analyses suggested moderate anecdotal evidence in support of the null hypothesis, that is, that no relationship existed between the Leisure subscale with Education (BF10 = 0.25) or Occupation (BF10 = 0.35). As such, these results suggest that our measure of cognitive reserve driving the associations with RT (i.e., the CRI Leisure subscale) is statistically independent of the education and occupation subscales. To address the concern that our observed associations between RT and CRI Leisure could be attributed to the effects of education or occupation, we reran the regression models while controlling for the effects of education and occupation. First, we reran the final regression model, in which response speed was modeled as a function of the CRI Leisure subscale, this time adding both the CRI Education and CRI Occupation subscales as nuisance covariates in step 1. With this approach, we observed that modeling RT as a function of CRI Education and CRI Occupation did not account for significant variance in RT (Adjusted R2 = 0.017, F(2,38) = 1.34, p = 0.27). Critically, when the CRI Leisure subscale was added to the next stage of the model, there was a significant improvement in model fit, over and above the null model ( [file ns-JN-RM-2260-21-s03.docx]

**Extended Data Figure 2-3. The effects of CRIq Leisure on RT are statistically independent to the association between RT and CRI Education and CRI Occupation**

|  | CRI *Leisure* | CRI *Education* | CRI *Occupation* | CRI *Composite* |
| --- | --- | --- | --- | --- |
| CRI *Leisure* |  |  |  |  |
| CRI *Education* | *r*=.11, *p*=.48  BF_10_=.25 |  |  |  |
| CRI *Occupation* | *r*=.18, *p*=.27  BF_10_=.35 | ***r*=.42, *p*=.006**  **BF_10_=7.14** |  |  |
| CRI  *Composite* | ***r*=.66, *p*<.001**  **BF_10_ = 8717.55** | ***r*=.62, *p*<.001**  **BF_10_=1698.38** | ***r*=.80, *p*<.001**  **BF_10_=2.161e+7** |  |

To explore the association between the CRIq Leisure subscale with Education and Occupation, we ran correlational analyses between the four CRI subscales (Extended Data Figure 2-3). First, the composite score was strongly associated with leisure, occupation, and education engagements (all *r*>.65), *p*<.001). A robust association was also observed between the CRI subscales of education and occupation (*r*>.4, see Table below). Critically, however, we did not find evidence to suggest an association existed between the CRI *Leisure* subscale with CRI Education (*r*=.11, *p*=.48) or CRI *Occupation* (*r*=.18, *p*=.27). In fact, follow up Bayesian analyses suggested moderate-anecdotal evidence in support of the null hypothesis, i.e., that no relationship existed between the Leisure subscale with Education (BF10=.25) or Occupation (BF10=.35). As such, these results suggest that our measure of cognitive reserve driving the associations with RT (i.e., the CRI leisure subscale) is statistically independent of the education and occupation subscales.

To address the concern that our observed associations between RT and CRI Leisure could be attributed to the effects of education or occupation, we re-ran the regression models while controlling for the effects of education and occupation.

First, we re-ran the final regression model, in which response speed was modelled as a function of the CRI Leisure subscale, this time adding both the CRI Education and CRI Occupation subscales as nuisance covariates in step 1. With this approach, we observed that modelling RT as a function of CRI Education and CRI Occupation did not account for significant variance in RT (Adjusted *R*^2^=.017, *F*_(2,38)_=1.34, *p*=.27). Critically, when the CRI Leisure subscale was added to the next stage of the model, there was a significant improvement in model fit, over and above the null model (Adjusted *R*^2=^.203, *R*^2^ change = .197, *F* change = 9.89, *F*_(3,37)_=4.40 , *p*=.01; Standardized *β* CRI Leisure= -0.45, *t*=-3.15, *p*=.003; 95% CI [-4.97 -1.07]; Standardized *β* CRI Education= 0.06, *t*=0.41,, *p*=.66; 95% CI [-2.71 4.08]; Standardized *β* CRI Occupation= .31, *t*=1.95, *p*=.06; 95% CI [-.07 3.91]).

Second, to verify our results using Bayesian statistics, we re-ran the Bayesian Linear Regression model, this time adding the CRI subscales of Education and Occupation to the null model, thereby exploring the direct association between the CRI Leisure and RT. This analysis indicated strong evidence in support of the hypothesis that CRI Leisure accounts for substantial variance in behavioural markers of response speed (BF_10_=14.38). As such, these results suggest our effects are driven by leisure activities, and not education or occupational engagements.
