# Supplementary figures and images for: Evidence Accumulation Rate Moderates the Relationship between Enriched Environment Exposure and Age-Related Response Speed Declines
Source: J Neurosci. 2023 Sep 13;43(37):6401–14. doi: 10.1523/JNEUROSCI.2260-21.2023 (PMC10500991; doi:10.1523/JNEUROSCI.2260-21.2023)

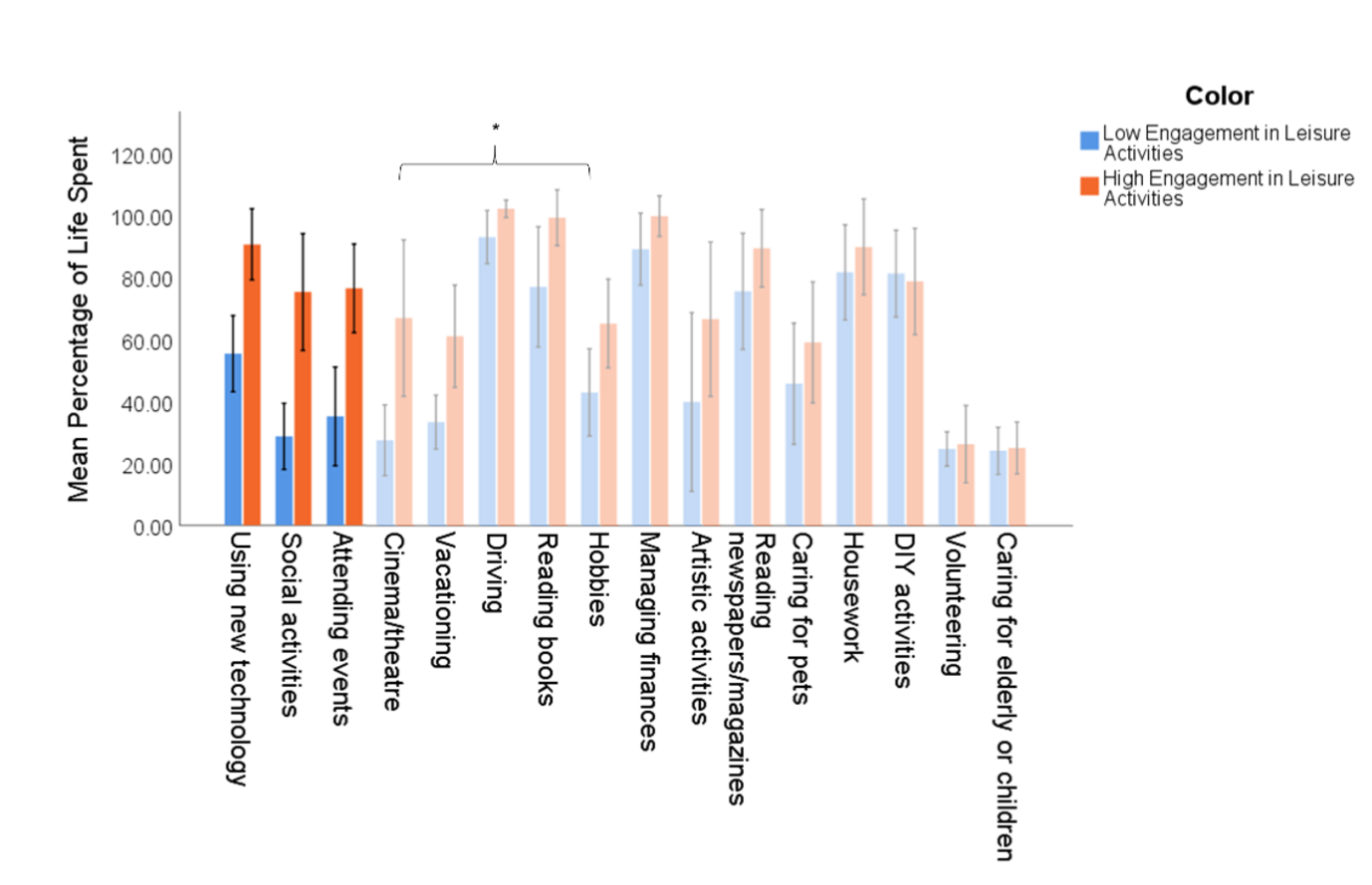

Supplement: Figure 2-4 — Differences between the activities of those with relatively higher versus lower levels of lifetime leisure engagement. Proportion of life spent engaging in particular activities varies between those with an overall higher or lower level of engagement in leisure activities. Significant group differences are presented in bold. The asterisk denotes comparisons where p < .05, but did not satisfy a Bonferroni-adjusted α = 0.003. To explore the leisure activities that may drive the apparent effect of EE on response speed, we compared the activities of those with higher versus lower levels of lifetime leisure engagement. To do so, we first devised two groups of older adults based on a median split of their engagement in leisure activities. Those with CRI Leisure subscores above the overall median score of 138.00 were considered High Engagement (n = 20), whereas those with a subscore equal to or lower than 138.00 were deemed Low Engagement (n = 21). We then examined each participant’s responses to individual activities on the CRIq. Participants first indicated whether they participated in the activity Often/Always, or Never/Rarely over the course of their lifetime and further specified for how many years they engaged Often/Always. For participants who engaged in an activity Often/Always for at least one year of life, we calculated separate values for their engagement in each activity, representing the percentage of life years spent engaging in each activity since 18 years of age using the following formula: [(Years of activity)/(Age – 18)] * 100. Note that rounding within the CRIq causes some individuals to exceed 100.00% of life spent participating in a given activity. For example, if an individual worked as a nurse for 3 years, this is rounded up to 5 years, as per the standardized questionnaire administration guidelines. Finally, we determined the percentage of individuals in each group who engaged in each activity Often/Always, and the mean percentage of life spent enga [file ns-JN-RM-2260-21-s04.tif]

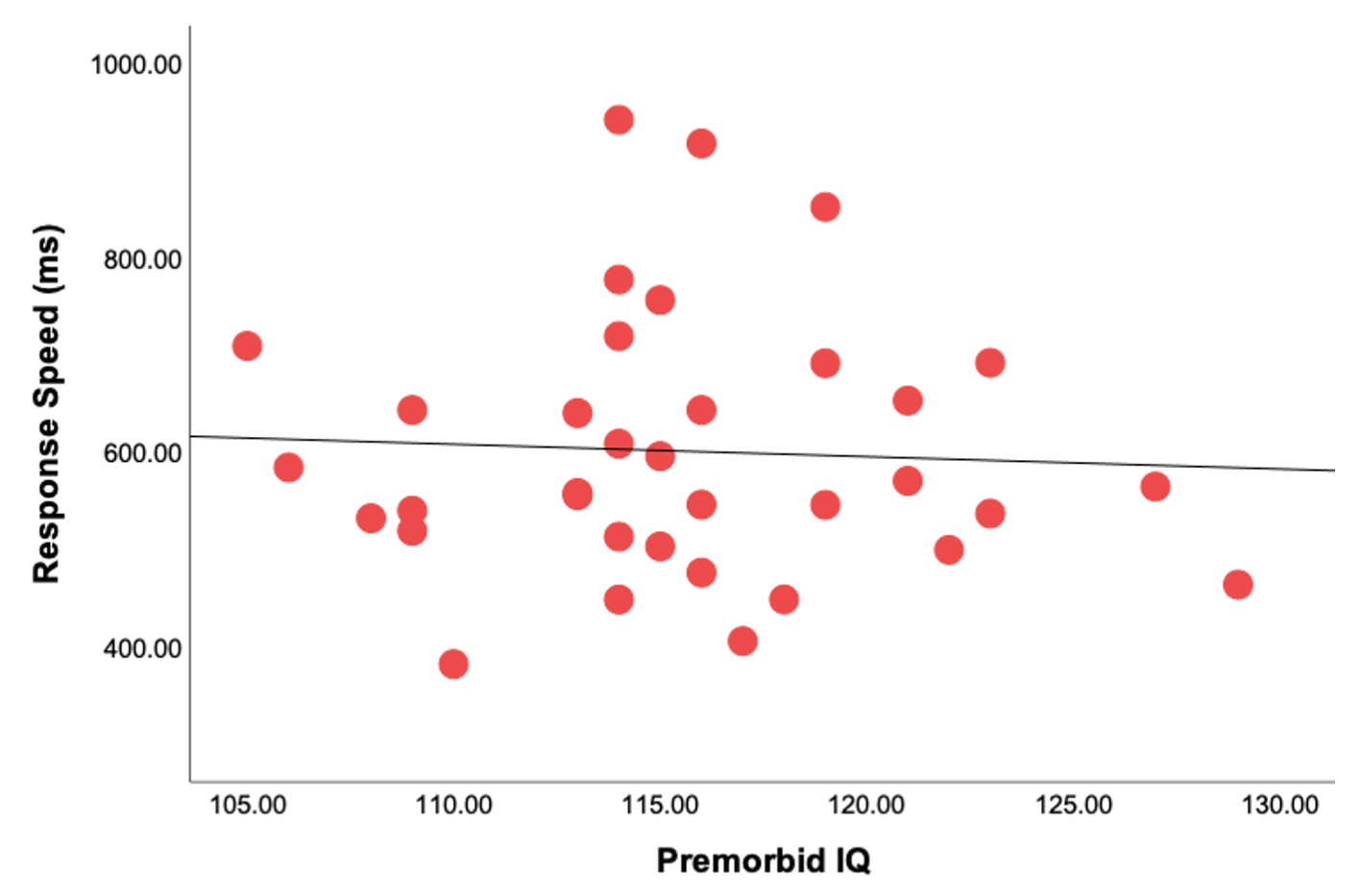

Supplement: Figure 2-5 — The impact of IQ on response speed. To investigate whether the observed relationship between EE and response speed could reflect individual differences in IQ, we estimated premorbid intelligence in a subset of participants (n = 36, data not shown). There was no direct association between IQ and response speed. Critically, the relationship between EE and response speed remained significant after covarying for IQ, indicating that the relationship between enrichment of cognition was not because of individual differences in intelligence. More specifically, a subset (N = 36) of the older adults completed word reading tasks commonly used to estimate premorbid IQ based on the Wechsler Adult Intelligence Scale–Fourth Edition (WAIS-IV; Wechsler, 1981). Of these individuals n = 17 completed the Test of Premorbid Function (ToPF), whereas the other n = 19 completed the National Adult Reading Test (NART; Nelson, 1982), using updated norms. Outliers were defined in SPSS using the IQR, consistent with the main analyses, separately for both cohorts of older adults. One outlier was detected for the ToPF and was subsequently removed and imputed using the mean value from their group. The two cohorts differed on estimates of IQ score derived using the different word lists (t(34) = 2.09, p = 0.04), with a subsequent Bayesian independent samples t test suggesting moderate evidence for a difference between the two groups, BF10 = 3.26. This is likely attributable to established differences in the estimations produced by the measure. Nonetheless, we considered it useful to investigate using the data available to us, whether our effects could be attributed to a relationship between response speed and IQ. For this, we ran a hierarchical linear regression of RT, with IQ entered as the first step in the model. IQ did not account for a statistically significant proportion of the variance in RT, indicating no direct influence of premorbid intelligence on response speed (F(1,34) = 0.07, p = 0.80, [file ns-JN-RM-2260-21-s05.tif]

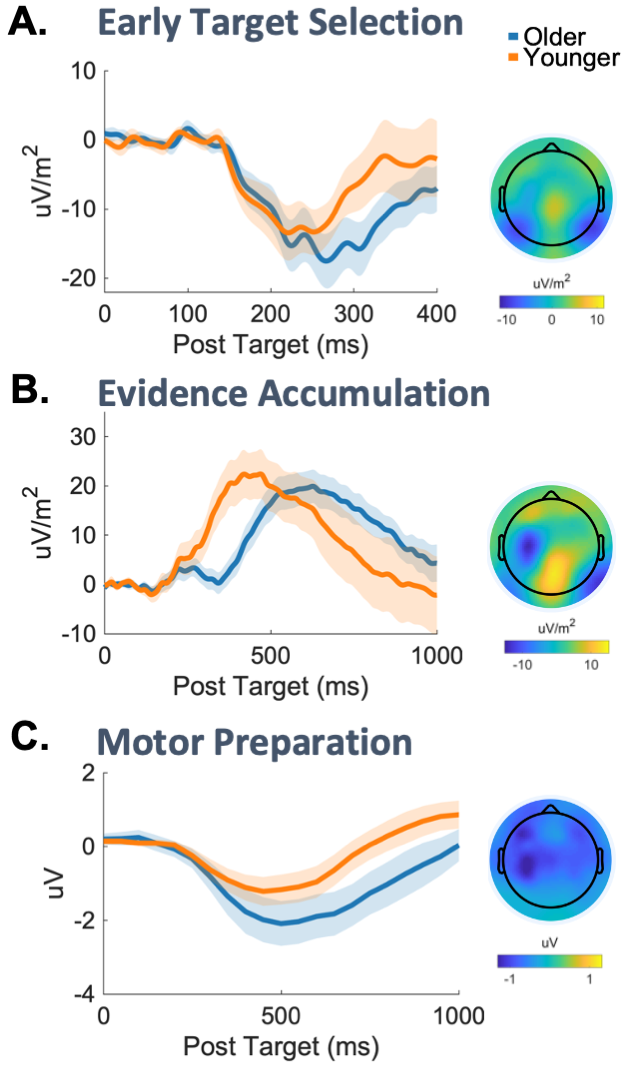

Supplement: Figure 3-2 — Temporal dynamics of evidence accumulation are robust age-related indicators A, The stimulus-aligned N2c waveform (electrodes P7/P8) for older and younger adults. B, Stimulus-aligned CPP waveform (electrode Pz) for the two groups. C, Stimulus-aligned beta waveform (electrode C3) for the two groups. The topoplots depict the spatial distribution of the EEG signal for both groups combined at 150–400 ms post-target for the N2c (A), −150 to 50 ms aligned to response for the CPP (B) and 400–700 ms post-target for LHB (C). We examined group-level differences in the eight electrophysiological markers using a series of one-way ANOVAs, Bonferroni-corrected for multiple comparisons (α 0.05/8 EEG components ≥ α-corrected threshold = 0.006) and supplemented these with Bayesian analyses to indicate the strength of evidence in support of the null hypothesis. No statistically significant difference was observed between older and younger adults in the latency of early target selection signals (N2c; F(1,70) = 2.75, p = 0.10, BF10 = 0.79; Extended Data Table 2-1; for plots and additional analyses see Extended Data Fig. 3-3), and although there was weak evidence to suggest that the amplitude of the N2c differed between groups (F(1,70) = 6.01, p = 0.02, partial η2 = 0.08, BF10 = 3.05; Extended Data Table 2-1, Extended Data Fig. 3-2A), this did not survive correction for multiple comparisons. In line with recent reports (McGovern et al., 2018), the older adults differed from their younger counterparts in metrics of evidence accumulation (the CPP). More specifically, timing delays were observed for several parameters of the CPP in older individuals; they showed a later onset (later CPP onset; F(1,70) = 14.8, p < 0.001, partial η2 = 0.18, BF10 = 96.52; Extended Data Table 2-1, Extended Data Fig. 3-2B) and slower build-up rate (shallower CPP build-up rate, slope; F(1,70) = 8.03, p = 0.006, partial η2 = 0.10, BF10 = 6.90; Extended Data Table 2-1 Extended Data Table 2-1, Extended Data Fig. 3- [file ns-JN-RM-2260-21-s07.tif]

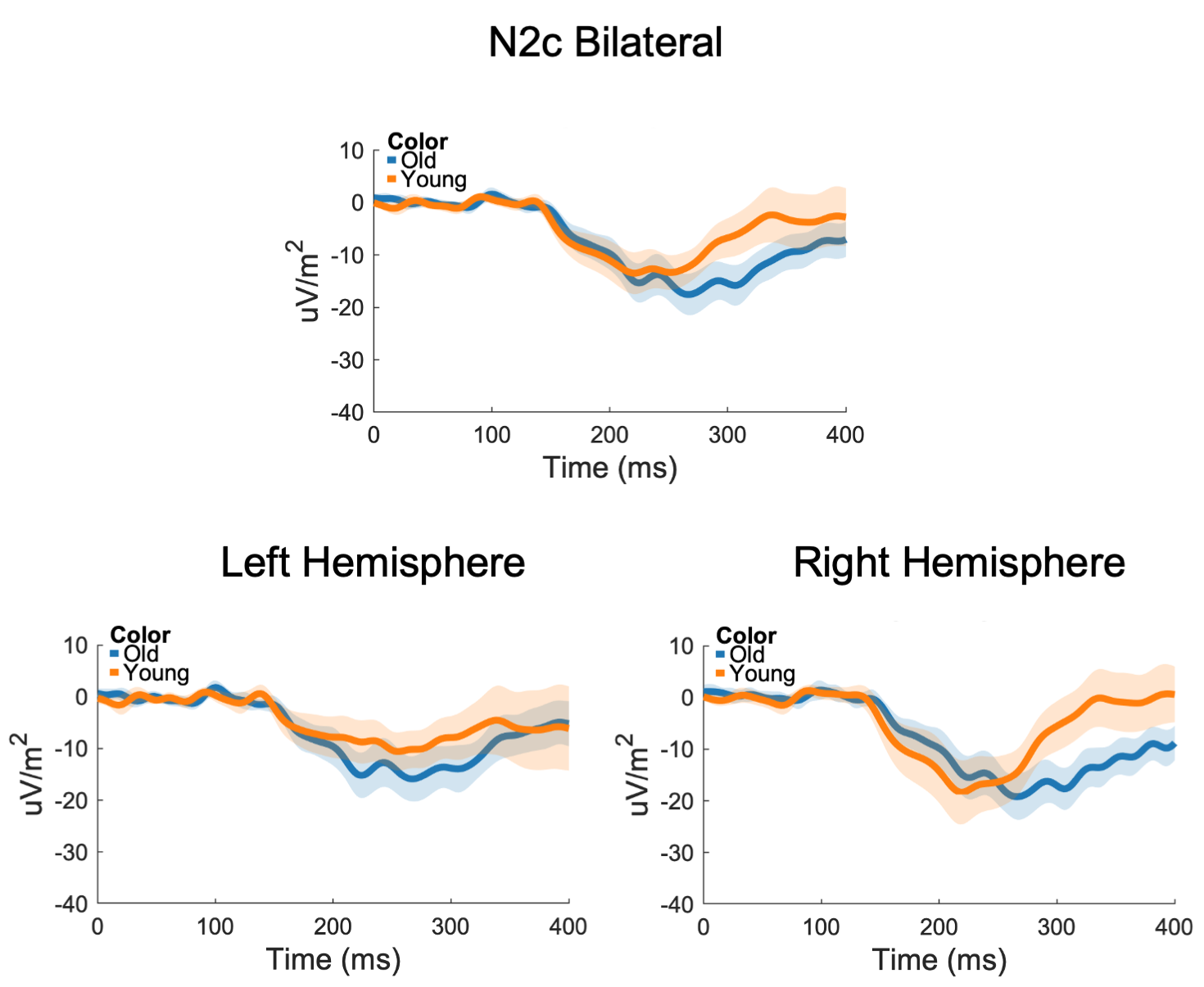

Supplement: Figure 3-3 — The N2c component, stimulus aligned at electrode P7 (left hemisphere), and P8 (right hemisphere). Given the relevance of hemisphere lateralization for theories of cognitive aging, we investigated any age-related hemisphere differences in the N2c using 2 (old vs young) × 2 (right hemisphere × left hemisphere) ANOVAs, separately for latency and amplitude. There was no main effect of hemisphere on N2c latency (F(1,69) = 3.46, p = 0.07) but there was a significant hemisphere × group interaction term (F(1,69) = 11.56, p = 0.001, partial η2 = 0.14). In line with a large body of work highlighting a right hemisphere dominance for early sensory processing, follow up analyses revealed that the younger adults showed a significantly faster right hemisphere N2c latency (mean = 257.07 ms, SD = 51.67) as compared with the left hemisphere (298.77 ms, 69.95; F(1,29) = 14.99, p = 0.001, partial η2 = 0.34). In contrast, for the older adults there was no hemispheric differences in N2c latency (F(1,40) = 1.23, p = 0.28; right hemisphere, mean = 303.59, SD = 57.56; left hemisphere, mean = 291.37 ms, SD = 54.86), possibly indicative of a reduction in hemispheric asymmetries in the older adults. As compared with the younger adults, the older adults showed slower N2c latencies in the right (F(1,69) = 12.32, p = 0.001, partial η2 = 0.15) but not left (F(1,69) = 0.25, p = 0.62) hemispheres. There was no effect of group on N2c amplitude (F(1,69) = 3.33, p = 0.07), nor was there any group × hemisphere interaction term (F(1,69) = 0.02, p = 0.88). Download Figure 3-3, TIF file. [file ns-JN-RM-2260-21-s08.tif]

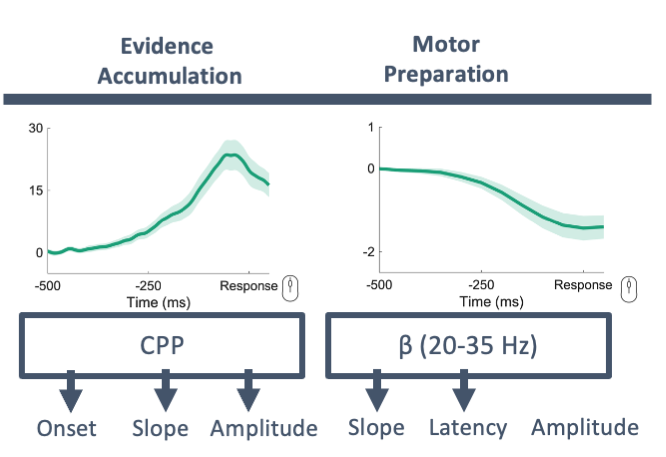

Supplement: Figure 3-4 — CPP and Beta components from Figure 1 visualized here aligned to participants’ response. To verify that motor preparatory activity was accurately captured by our stimulus-locked measure of beta latency, and to exclude the possibility that EE could be affecting RT through an influence over motor preparatory activity, two response-locked beta metrics were derived and explored in relation to RT, response-locked beta slope (build-up rate) and response-locked beta amplitude (threshold). Beta slope was defined as the slope of a straight line fitted to the response- locked waveform, with the time window defined individually for each participant between 300 to 50 ms preresponse, and baselined to −450 to −350 ms. Beta amplitude was measured as the mean amplitude of a 100 ms window centered on a participants’ response (i.e., −50 to +50 ms around response). A stepwise linear regression model was used to identify which of the three beta measures (peak stimulus-locked latency, along with slope, and amplitude at the time of response) was the best predictor of RT (criteria, probability of F to enter ≤ 0.05, probability of F to remove ≤ 0.1). The resulting model of RT included only stimulus-locked beta latency, indicating that this was the most appropriate EEG metric for capturing independent variance in RT (beta latency, standardized β = 0.54, t = 5.25, p < 0.001, 95% CI, 0.28, 0.62; beta slope: β = 0.10, t = 0.95, p = 0.34, beta amplitude β = − 0.12, t = −1.14, p = 0.26, model F(1,68) = 27.60, p < 0.001). In line with previous work (O’Connell et al., 2012; Brosnan et al., 2020), this result suggests that beta latency is a valid marker of task-relevant motor preparatory activity accounting for independent variance in response speed. Download Figure 3-4, TIF file. [file ns-JN-RM-2260-21-s09.tif]

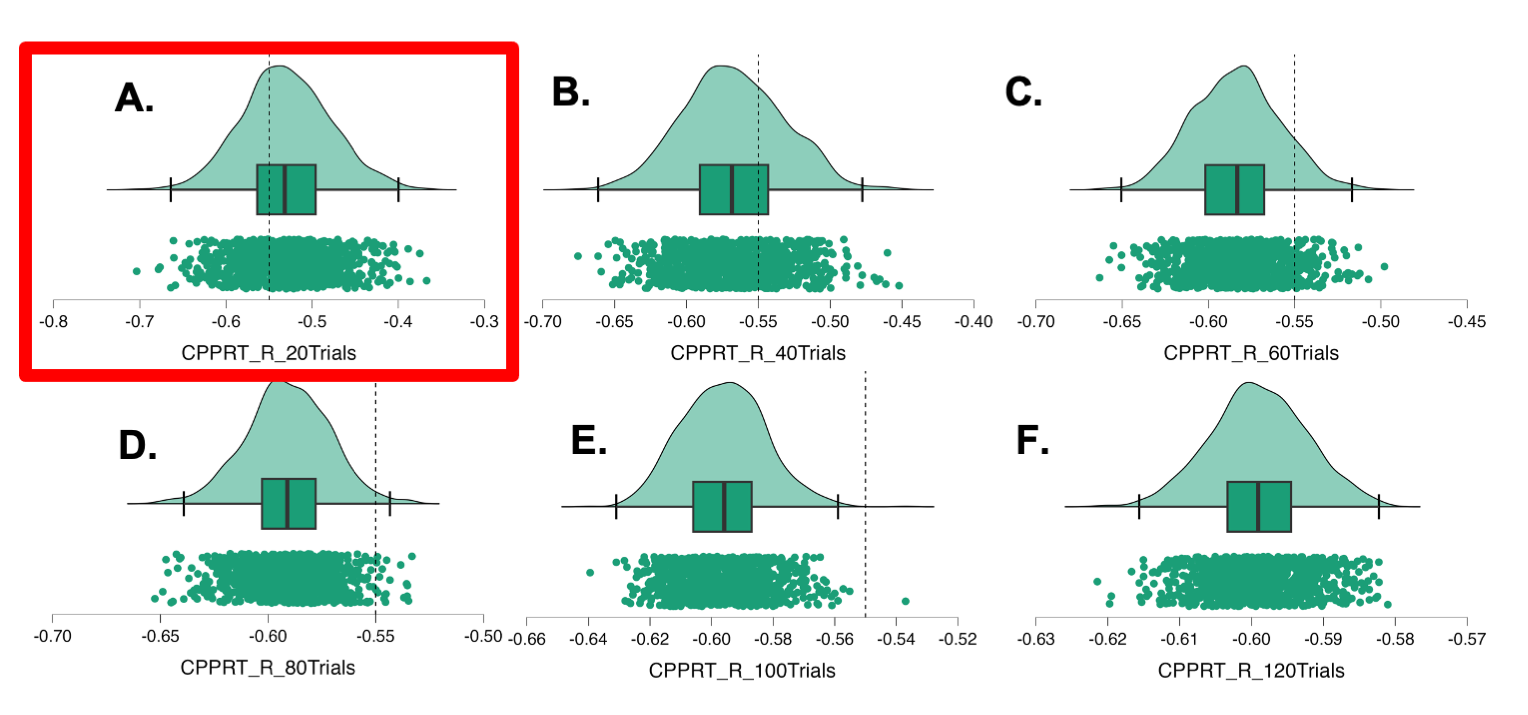

Supplement: Figure 5-2 — A–F, Each subplot denotes the direct relationship, Pearson’s R, between CPP build-up rate and RT for each of the 1000 permutations for each of the six bin sizes (20 up until 120 trials). Note the x-axis scales vary according to subplots. The dotted lines indicate a Pearson’s r value of −0.55 between CPP build-up rates and RTs. Bayesian analyses indicate strong/infinite support for the alternative hypothesis that the effect sizes for the relationship between RT and CPP build-up rate with 120, 100, 80, 60, and 40 trials were larger than 0.55 (i.e., less than 0.55 given the negative relationship between CPP build-up rate and response speed; all BF10 > 2.314 × 10+63). However, Bayes factor analyses revealed strong support for the null hypothesis (BF10 = 0.002), that is, that the estimates of effect size were not greater than 0.55 (A, red). Calculation of the time necessary to assess 40 trials of the CPP. Results from the minimum trial analyses indicate that a minimum of 40 trials would be sufficient to derive valid and behaviorally meaningful estimates of CPP build-up rate. These trials are derived using the response-locked EEG signals, following data cleaning, for correctly identified target stimuli (coherently moving dots). Below we provide calculations both for the average time we expect necessary to obtain 40 valid trials, and for a worst case scenario. Calculations using mean values. Mean accuracy was 96%. To get 40 valid response locked trials, participants would need to be administered an extra 4% (2 trials), that is, 42 trials in total. We calculated the percentage of EEG trials that were rejected by data cleaning, that is, [rejected trials/(rejected trials + valid trials], and on average 15% of trials were excluded following the preprocessing steps. In order to obtain 42 valid EEG trials (after data cleaning), an additional 15% of data would need to be collected, so 48 trials in total. in this case, we would present the participant with 48 coherent motion trial [file ns-JN-RM-2260-21-s11.tif]
