# Supplementary material for: Evidence Accumulation Rate Moderates the Relationship between Enriched Environment Exposure and Age-Related Response Speed Declines
Source: J Neurosci. 2023 Sep 13;43(37):6401–14. doi: 10.1523/JNEUROSCI.2260-21.2023 (PMC10500991; doi:10.1523/JNEUROSCI.2260-21.2023)
Supplement: Figure 3-1 — Bayesian linear regression model statistics examining how each neurophysiological marker contributed to RT. Note. BFinclusion values above one indicate the strength of evidence in favor of the alternative hypothesis and are highlighted in bold. Download Figure 3-1, DOCX file. [file ns-JN-RM-2260-21-s06.docx]

**Extended Data Figure 3-1:** Bayesian linear regression model statistics examining how each neurophysiological marker contributed to RT. Note. BF_inclusion_ values above 1 indicate the strength of evidence in favour of the alternative hypothesis and are highlighted in bold.

| **Coefficient** | | **BF_inclusion_ (BF_10_)** | | **Mean** | | **SD** | | **95% Credible Interval** | | | |  |
| --- | --- | --- | --- | --- | --- | --- | --- | --- | --- | --- | --- | --- |
| Intercept |  |  | 1.000 |  | 527.214 |  | 9.128 |  | 508.296 |  | 544.450 |  |
| **Age** |  |  | **1028.523** |  | **2.014** |  | **0.471** |  | **1.083** |  | **2.967** |  |
| N2c Latency |  |  | 0.728 |  | 0.082 |  | 0.155 |  | -0.058 |  | 0.512 |  |
| N2c Amplitude |  |  | 0.619 |  | -0.275 |  | 0.698 |  | -2.431 |  | 0.743 |  |
| CPP Onset |  |  | 0.545 |  | 0.020 |  | 0.068 |  | -0.105 |  | 0.205 |  |
| **CPP Build-Up Rate** |  |  | **4732.187** |  | **-889.868** |  | **189.103** |  | **-1298.926** |  | **-542.033** |  |
| **CPP Amplitude** |  |  | **17.670** |  | **2.586** |  | **1.109** |  | **0.000** |  | **4.319** |  |
| LHB Build-Up Rate |  |  | 0.683 |  | 919.718 |  | 1914.117 |  | -1776.934 |  | 6581.070 |  |
| **LHB Latency** |  |  | **31.742** |  | **0.230** |  | **0.083** |  | **0.000** |  | **0.365** |  |
| LHB Amplitude |  |  | 0.750 |  | -4.347 |  | 8.050 |  | -23.447 |  | 6.318 |  |
