# Supplementary material for: Evidence Accumulation Rate Moderates the Relationship between Enriched Environment Exposure and Age-Related Response Speed Declines
Source: J Neurosci. 2023 Sep 13;43(37):6401–14. doi: 10.1523/JNEUROSCI.2260-21.2023 (PMC10500991; doi:10.1523/JNEUROSCI.2260-21.2023)
Supplement: Table 2-1 — All values signify mean (M) and SD) EEG signals that differed significantly between the older and younger adults (according to both Bonferroni-corrected frequentist and Bayesian analyses) are highlighted in bold. Weak (anecdotal) evidence is provided for the group difference in N2c amplitude. Download Table 2-1, DOCX file. [file ns-JN-RM-2260-21-s17.docx]

**Extended Data Table 2-1. *Note* all values signify mean (M) and standard deviations (SD). EEG signals that differed significantly between the older and younger adults (according to both Bonferroni-corrected frequentist and Bayesian analyses) are highlighted in bold. *Note*, weak (anecdotal) evidence is provided for the group difference in N2c amplitude**

|  | Younger Adults (*N*=31) | Older Adults (*N*=41) |
| --- | --- | --- |
| N2c Amplitude (uV/m^2^) | -10.03 (8.35) | -15.98 (11.36) |
| N2c Latency (ms) | 266.16 (55.61) | 285.78 (44.81) |
| CPP Onset (ms) | 250.84 (80.39) | 345.17 (117.16) |
| CPP Build-up Rate | .12 (.07) | .07 (.07) |
| CPP Amplitude (uV/m^2^) | 24.32 (13.75) | 17.20 (11.16) |
| LHB Latency (ms) | 471.58 (98.18) | 549.02 (178.46) |
| LHB Slope | -.0059 (.0057) | -.0068 (.0067) |
| LHB Amplitude | -1.32 (1.26) | -2.04 (1.73) |
