# Supplementary material for: Evidence Accumulation Rate Moderates the Relationship between Enriched Environment Exposure and Age-Related Response Speed Declines
Source: J Neurosci. 2023 Sep 13;43(37):6401–14. doi: 10.1523/JNEUROSCI.2260-21.2023 (PMC10500991; doi:10.1523/JNEUROSCI.2260-21.2023)
Supplement: Table 2-2 — Hierarchical linear regression model statistics examining how each neurophysiological marker contributed to RT, over and above the contributions made by those processes that temporally preceded. Download Table 2-2, DOCX file. [file ns-JN-RM-2260-21-s18.docx]

**Extended Data Table 2-2.** Hierarchical linear regression model statistics examining how each neurophysiological marker contributed to RT, over and above the contributions made by those processes that temporally preceded.

| **Hierarchical Modelling of RT** | | | | | | |
| --- | --- | --- | --- | --- | --- | --- |
| Model | | Sum of Squares | df | Mean Square | F | Sig. |
| **Age (centred)** | |  |  |  |  |  |
| 1 | Regression | 440379.981 | 1 | 440379.981 | 40.705 | .000^b^ |
|  | Residual | 746497.607 | 69 | 10818.806 |  |  |
|  | Total | 1186877.588 | 70 |  |  |  |
| **+ N2c Latency** | |  |  |  |  |  |
| 2 | Regression | 457738.096 | 2 | 228869.048 | 21.344 | .000^c^ |
|  | Residual | 729139.492 | 68 | 10722.640 |  |  |
|  | Total | 1186877.588 | 70 |  |  |  |
| **+ N2c Amplitude** | |  |  |  |  |  |
| 3 | Regression | 474126.961 | 3 | 158042.320 | 14.856 | .000^d^ |
|  | Residual | 712750.627 | 67 | 10638.069 |  |  |
|  | Total | 1186877.588 | 70 |  |  |  |
| **+ CPP Onset Latency** | | |  |  |  |  |
| 4 | Regression | 477414.083 | 4 | 119353.521 | 11.103 | .000^e^ |
|  | Residual | 709463.505 | 66 | 10749.447 |  |  |
|  | Total | 1186877.588 | 70 |  |  |  |
| **+ CPP Build-Up Rate** | | |  |  |  |  |
| 5 | Regression | 675255.405 | 5 | 135051.081 | 17.158 | .000^f^ |
|  | Residual | 511622.183 | 65 | 7871.111 |  |  |
|  | Total | 1186877.588 | 70 |  |  |  |
| **+ CPP Amplitude** | | |  |  |  |  |
| 6 | Regression | 757783.795 | 6 | 126297.299 | 18.837 | .000^g^ |
|  | Residual | 429093.793 | 64 | 6704.591 |  |  |
|  | Total | 1186877.588 | 70 |  |  |  |
| **+ LHB Build-Up Rate** | | |  |  |  |  |
| 7 | Regression | 757824.042 | 7 | 108260.577 | 15.896 | .000^h^ |
|  | Residual | 429053.546 | 63 | 6810.374 |  |  |
|  | Total | 1186877.588 | 70 |  |  |  |
| **+ LHB Peak Latency** | | |  |  |  |  |
| 8 | Regression | 816914.302 | 8 | 102114.288 | 17.113 | .000^i^ |
|  | Residual | 369963.286 | 62 | 5967.150 |  |  |
|  | Total | 1186877.588 | 70 |  |  |  |
| **+ LHB Amplitude** | | |  |  |  |  |
| 9 | Regression | 828221.160 | 9 | 92024.573 | 15.651 | .000^j^ |
|  | Residual | 358656.428 | 61 | 5879.614 |  |  |
|  | Total | 1186877.588 | 70 |  |  |  |
