# Supplementary material for: Evidence Accumulation Rate Moderates the Relationship between Enriched Environment Exposure and Age-Related Response Speed Declines
Source: J Neurosci. 2023 Sep 13;43(37):6401–14. doi: 10.1523/JNEUROSCI.2260-21.2023 (PMC10500991; doi:10.1523/JNEUROSCI.2260-21.2023)
Supplement: Figure 5-1 — Extended Data. Download Figure 5-1, DOCX file. [file ns-JN-RM-2260-21-s10.docx]

Extended Data Figure 5-1.

|  | 120 vs 100 | 100 vs 80 | 80 vs 60 | 60 vs 40 | 40 vs 20 |
| --- | --- | --- | --- | --- | --- |
| Cumulative Distribution Factor (ks) | | | | | |
| RT (*k, p*) | ***k*=.16***** | ***k* =.10***** | ***k*=.09***** | ***k*=.11**** | ***k*=.09***** |
| CPP slope | ***k*=.17***** | ***k*=.08**** | ***k*=.08**** | ***k*=.08**** | ***k*=.12***** |

*Note*. ks denotes Kolmogorov-Smirnov test, *****, *p<*.001, **, *p*<.01**
