# Supplementary material for: Evidence Accumulation Rate Moderates the Relationship between Enriched Environment Exposure and Age-Related Response Speed Declines
Source: J Neurosci. 2023 Sep 13;43(37):6401–14. doi: 10.1523/JNEUROSCI.2260-21.2023 (PMC10500991; doi:10.1523/JNEUROSCI.2260-21.2023)
Supplement: Figure 6-1 — The t parameter (nondecision time) modeled using a hierarchical linear regression model as a function of the EEG metrics. Download Figure 6-1, DOCX file. [file ns-JN-RM-2260-21-s12.docx]

**Extended Data Figure 6-1.** ***t* parameter (non-decision time) modelled using a hierarchical linear regression model as a function of the EEG metrics.**

| Model | *R*^2^ | Adj *R*^2^ | *F* Change | Sig *F* Change |
| --- | --- | --- | --- | --- |
| A | 0.003 | -0.011 | 0.227 | 0.635 |
| B | 0.049 | 0.021 | 3.255 | 0.076 |
| C | 0.053 | 0.01 | 0.262 | 0.61 |
| D | 0.088 | 0.033 | 2.573 | 0.113 |
| E | 0.127 | 0.06 | 2.914 | 0.093 |
| F | 0.141 | 0.06 | 1.014 | 0.318 |
| G | 0.145 | 0.05 | 0.309 | 0.58 |

***Note*.** Note each EEG signal is added sequentially in a hierarchical manner based on the temporal order in which they occur. Each model includes the addition of: **A.** Age. **B.** N2c Amplitude **C**. N2c Latency **D**. CPP onset **E.** CPP build-up rate **F.** CPP Amplitude **G.** LHB Peak Latency.
