# Supplementary material for: Evidence Accumulation Rate Moderates the Relationship between Enriched Environment Exposure and Age-Related Response Speed Declines
Source: J Neurosci. 2023 Sep 13;43(37):6401–14. doi: 10.1523/JNEUROSCI.2260-21.2023 (PMC10500991; doi:10.1523/JNEUROSCI.2260-21.2023)
Supplement: Figure 6-2 — ν parameter (drift rate) modeled using a hierarchical linear regression model as a function of the EEG metrics. Download Figure 6-2, DOCX file. [file ns-JN-RM-2260-21-s13.docx]

**Extended Data Figure 6-2. *ν* parameter (drift rate) modelled using a hierarchical linear regression model as a function of the EEG metrics.**

| Model | *R*^2^ | Adj *R*^2^ | *F* Change | Sig *F* Change |
| --- | --- | --- | --- | --- |
| ***Ν* parameter (drift rate)** | | | | |
| **A** | **0.077** | **0.063** | **5.741** | **0.019*** |
| B | 0.077 | 0.05 | 0.006 | 0.94 |
| C | 0.077 | 0.036 | 0.002 | 0.962 |
| D | 0.085 | 0.03 | 0.594 | 0.444 |
| **E** | **0.148** | **0.082** | **4.764** | **0.033*** |
| F | 0.154 | 0.075 | 0.481 | 0.49 |
| G | 0.179 | 0.088 | 1.914 | 0.171 |

***Note*.** Note each EEG signal is added sequentially in a hierarchical manner based on the temporal order in which they occur. Each model includes the addition of: **A.** Age. **B.** N2c Amplitude **C**. N2c Latency **D**. CPP onset **E.** CPP build-up rate **F.** CPP Amplitude **G.** LHB Peak Latency. * denotes a significant change in model fit.
