# Supplementary material for: Evidence Accumulation Rate Moderates the Relationship between Enriched Environment Exposure and Age-Related Response Speed Declines
Source: J Neurosci. 2023 Sep 13;43(37):6401–14. doi: 10.1523/JNEUROSCI.2260-21.2023 (PMC10500991; doi:10.1523/JNEUROSCI.2260-21.2023)
Supplement: Figure 6-3 — Final model of ν parameter (drift rate) as a function of the EEG metrics. Download Figure 6-3, DOCX file. [file ns-JN-RM-2260-21-s14.docx]

**Extended Data Figure 6-3. Final model of *ν* parameter (drift rate) as a function of the EEG metrics.**

|  | Standardised β | *t* | *p* | 95% CI |
| --- | --- | --- | --- | --- |
| Age | -.2 | -1.57 | .12 | [-0.4 .00] |
| **CPP build-up rate** | **.28** | **2.34** | **.02** | **[1.15 14.60]** |

*Note*. This model explained 12% of the variance in drift rate (ν; *F*_2,68_=5.78, *p*<.005)
