# Supplementary material for: Evidence Accumulation Rate Moderates the Relationship between Enriched Environment Exposure and Age-Related Response Speed Declines
Source: J Neurosci. 2023 Sep 13;43(37):6401–14. doi: 10.1523/JNEUROSCI.2260-21.2023 (PMC10500991; doi:10.1523/JNEUROSCI.2260-21.2023)
Supplement: Figure 6-4 — a parameter (response caution) modeled using a hierarchical linear regression model as a function of the EEG metrics. Download Figure 6-4, DOCX file. [file ns-JN-RM-2260-21-s15.docx]

**Extended Data Figure 6-4. *a* parameter (response caution) modelled using a hierarchical linear regression model as a function of the EEG metrics.**

| Model | *R*^2^ | Adj *R*^2^ | *F* Change | Sig *F* Change |
| --- | --- | --- | --- | --- |
| ***a* parameter (response caution)** | | | | |
| **A** | **0.093** | **0.08** | **7.06** | **0.01*** |
| B | 0.093 | 0.067 | 0.03 | 0.864 |
| C | 0.107 | 0.067 | 1.045 | 0.31 |
| D | 0.129 | 0.076 | 1.624 | 0.207 |
| E | 0.132 | 0.065 | 0.266 | 0.608 |
| **F** | **0.215** | **0.141** | **6.739** | **0.012*** |
| **G** | **0.297** | **0.219** | **7.387** | **0.008*** |

***Note*.** Note each EEG signal is added sequentially in a hierarchical manner based on the temporal order in which they occur. Each model includes the addition of: **A.** Age. **B.** N2c Amplitude **C**. N2c Latency **D**. CPP onset **E.** CPP build-up rate **F.** CPP Amplitude **G.** LHB Peak Latency. * denotes a significant change in model fit.
