# Supplementary material for: Evidence Accumulation Rate Moderates the Relationship between Enriched Environment Exposure and Age-Related Response Speed Declines
Source: J Neurosci. 2023 Sep 13;43(37):6401–14. doi: 10.1523/JNEUROSCI.2260-21.2023 (PMC10500991; doi:10.1523/JNEUROSCI.2260-21.2023)
Supplement: Figure 6-5 — Final model of a parameter (response caution) as a function of the EEG metrics. Download Figure 6-5, DOCX file. [file ns-JN-RM-2260-21-s16.docx]

**Extended Data Figure 6-5. Final model of *a* parameter (response caution) as a function of the EEG metrics.**

|  | Standardised β | *t* | *p* | 95% CI |
| --- | --- | --- | --- | --- |
| Age | .24 | 2.09 | .04 | [.001 .03] |
| CPP amplitude | .20 | 1.83 | .07 | [-.002 .05] |
| LHB latency | .38 | 3.35 | .001 | [.001 .006] |

***Note*.** This model explained 21.7% of the variance in response caution (*a*; *F*_3,67_=7.46, *p*<.001)
